# Cardioneuroablation Acutely Affects the Amplitude and Efficiency of Respiratory Heart Rate Variability

**DOI:** 10.3390/jcm15010382

**Published:** 2026-01-05

**Authors:** Piotr Niewinski, Stanislaw Tubek, Krzysztof Nowak, Krystian Josiak, Bartłomiej Paleczny

**Affiliations:** 1 Institute of Heart Diseases, Faculty of Medicine, Wroclaw Medical University, 50-556 Wroclaw, Poland; stanislaw.tubek@umw.edu.pl (S.T.); krzysztof.nowak@umw.edu.pl (K.N.); 2 Clinical Department of Cardiology, Jan Mikulicz-Radecki University Hospital, 50-556 Wroclaw, Poland; 3 Department of Cardiology, 4th Military Hospital, 50-981 Wroclaw, Poland; elektrofizjologia@4wsk.pl; 4 Department of Physiology and Pathophysiology, Wroclaw Medical University, 50-386 Wroclaw, Poland; bartlomiej.paleczny@umw.edu.pl

**Keywords:** baroreflex, cardioneuroablation, cardiorespiratory coupling, heart rate, parasympathetic system, respiratory sinus arrhythmia, respiratory heart rate variability

## Abstract

**Background/Objectives**: Cardioneuroablation (CNA) is used to treat reflex syncope by parasympathetic denervation of the cardiac conduction system. Respiratory heart rate variability (RespHRV) constitutes an important physiological mechanism that optimizes lung perfusion. The impact of CNA on various components of RespHRV remains unclear. **Methods**: Eleven subjects (36.8 ± 14.1 years) undergoing CNA for the treatment of cardioinhibitory, vagally mediated syncope were enrolled. For the RespHRV assessment, we used continuous respiratory flow measurement and an electrocardiogram. RespHRV analysis included the following: (a) amplitude, reflecting the overall magnitude of changes in RR interval during the respiratory cycle (RespHRVpv, ms); and (b) efficiency, defined as the percentage of inspirations accompanied by RR shortening (short-RRi inspirations, %), and expirations accompanied by RR prolongation (long-RRi expirations, %). Baroreflex sensitivity (BRS, ms/mmHg) was assessed with a sequential method using a noninvasive hemodynamic monitor. Both RespHRV and BRS were captured 48 h apart, before and after CNA. **Results**: A significant reduction was observed in RespHRVpv (57 [30–131] vs. 13 [7–16] ms, *p* = 0.003), short-RRi inspirations (97.0 [77.8–100.0] vs. 36.0 [14.3–63.2] %, *p* = 0.003), and long-RRi expirations (88.0 [78.1–97.6] vs. 31.1 [21.4–65.8] %, *p* = 0.008). Moreover, we found a strong relationship between ΔBRS and ΔRespHRVpv (r = 0.77, *p* = 0.005) following CNA. **Conclusions**: Our results indicate a substantial role of the cardiac parasympathetic system in RespHRV development, including both its amplitude and efficiency. The marked decrease in key RespHRV measures after CNA highlights the need for further research into its long-term clinical effects.

## 1. Introduction

Cardioneuroablation (CNA) has recently become a treatment option for neurogenic syncope [1]. CNA significantly affects heart rate variability, baroreflex function, and hypoxic reactivity of the sinus node [2,3]. The observed decrease in high-frequency (HF) power may indicate diminished heart rate (HR) modulation in response to breathing patterns [4]; however, this has not yet been examined through simultaneous recordings of HR and respiratory flow.

The CNA model offers a unique opportunity to investigate the role of parasympathetic innervation of the heart in the physiology of respiratory heart rate variability (RespHRV). This significant phenomenon—characterized by an increase in HR during inspiration and a decrease during expiration—has been observed in all free-breathing vertebrates, and, as elegantly demonstrated by Hayano et al. [5,6], was found to enhance gas exchange efficiency. It was previously named “respiratory sinus arrhythmia”, but because of misleading, negative connotations, the new term (RespHRV) has been recently proposed [7]. We hypothesize that the parasympathetic fibers innervating the sinus node primarily participate in the neural signaling responsible for HR modulation during respiration. This hypothesis could be tested by evaluating RespHRV indices before and after the CNA procedure, which specifically targets vagal innervation of the sinus node. Additionally, an important unanswered question is whether the intact parasympathetic system of the heart controls both the amplitude of RespHRV (the extent of HR variation between inspiration and expiration) and its efficiency (the degree of synchronization between HR changes and specific respiratory phases), or if the latter relies on mechanisms other than vagal transmission.

The specific neural pathway responsible for the origin of RespHRV (whether baroreflex-mediated or centrally driven) remains unclear [8,9]. In addition to autonomic neural control, the stretching of the sinus node caused by changes in intrathoracic pressure has also been proposed to play a role in maintaining RespHRV [10]. Perhaps, by examining the potential relationship between changes in RespHRV indices and alterations in baroreflex gain after CNA, we could gain new insights into this debated subject.

Disruption of RespHRV following CNA could also be of clinical importance, given the fact that approximately 12% of patients report worsening of exercise capacity after the procedure [11]. Recognizing studies that associate better physical performance with greater levels of cardiorespiratory coupling [12,13], one might speculate that reduced RespHRV could contribute to the pathophysiology of this specific adverse effect of CNA. Certainly, describing changes in RespHRV after CNA would add an essential perspective to the discussion about the advantages and disadvantages of parasympathetic denervation in humans.

## 2. Materials and Methods

This study’s protocol was approved by the local Institutional Ethics Committee (approval no. KB-331/2022) and followed the standards of the Declaration of Helsinki. Informed consent was obtained in writing from all study participants. This study is registered with ClinicalTrials.gov (NCT06697145).

### 2.1. Participating Criteria and Study Protocol

We studied patients eligible for CNA as a treatment for reflex syncope who presented with sinus rhythm. Individuals with structural heart disease or on chronic pharmacological treatment interfering with HR were excluded. The analysis of RespHRV and baroreflex function was performed one day before and one day after CNA (approximately 48 h apart), using resting recordings of electrocardiogram (ECG), blood pressure, and respiratory airflow, as explained below. The same 180-second-long recording was used for all analyses. Testing was performed in a supine position in a quiet, light-attenuated room at a stable temperature of 22–24 °C, preceded by sufficient time for familiarization with the study equipment.

### 2.2. Data Processing and Statistical Analysis

LabChart v. 8 (ADInstruments, Sydney, Australia) and MATLAB 2021b (Mathworks, Natick, MA, USA) were used for data processing. Statistica v.13.3 (StatSoft, Tulsa, OK, USA) was used for statistical analysis. Data are presented as median with lower and upper quartiles or mean with standard deviation, where appropriate. Wilcoxon’s test, Student’s paired t-test, and Pearson’s r correlation coefficient were used. A *p*-value < 0.05 was considered statistically significant.

#### 2.2.1. ECG and Blood Pressure Data

The R-wave timestamps were marked on the ECG signal using the Cyclic Measurements module of LabChart v. 8 (ADInstruments, Sydney, Australia) and exported at the original sampling rate (1000 Hz) to a text file. Then, a custom MATLAB script identified ectopic beats based on RRi lengths below a threshold of 0.55 s and corrected them through simple interpolation, relocating the ectopic beat to halfway between the adjacent regular beats. The value of systolic blood pressure (SBP) was extracted for every RRi using Finapres technology (FMS, Enschede, The Netherlands) and a MATLAB script.

#### 2.2.2. Respiratory Data

All respiratory data were derived from the expiratory airflow measured with a differential pressure transducer and a respiratory flowhead (ADInstruments, Sydney, Australia). The raw signal was integrated during each expiration, with the integration reset when the airflow returned to zero. This produced a series of ramp-like waveforms: expirations—characterized by a gradual rise and an abrupt drop to zero (end of expiration)—interspersed with periods of no airflow, which correspond to inspirations. The Cyclic Measurements module of the LabChart v. 8 (ADInstruments, Sydney, Australia) was used to mark the start of each inspiration (the last non-zero sample at the end of expiration) and expiration (when expiratory airflow rose above the threshold of 0.05 L) on the integrated signal. The respiratory data were exported at the original sampling rate (1000 Hz) to a text file for further processing with a custom script written in MATLAB 2021b. MATLAB processing included the following: (1) verifying the alternating occurrence of inspirations and expirations—if no or multiple expiration markers were found between two subsequent inspirations, the existing markers were removed, and a new marker was placed halfway between the inspirations; (2) automatically removing the 5% shortest and longest breaths. Finally, the original recordings and processed data were visually inspected (B.P.) to verify the accuracy of the script-based analysis.

### 2.3. Respiratory Heart Rate Variability Assessment

RespHRV was evaluated based on (1) amplitude (inspiratory–expiratory difference in RRi length) and (2) efficiency (the percentage of respiratory events associated with RespHRV-specific changes in RRi length).

RespHRV amplitude was measured using the well-established peak–valley method [14,15]. Details of the computational process were adapted from Goedhart et al. [16] with minor modifications. In the peak–valley method, for each respiratory cycle, the shortest RRi within the inspiratory window is identified and subtracted from the longest RRi within the expiratory window. The search window for the shortest RRi during inspiration and the longest RRi during expiration was shifted forward in relation to respiratory markers to account for a physiological delay between respiration and cardiac response. Unlike Goedhart et al. [16], this delay was not fixed but was adjusted individually based on the time from the onset of inspiration to the first RRi shorter than the previous one. This time span was calculated over the first five respiratory cycles, and the average value was used as the individually adjusted RespHRV delay (s). The inspiratory–expiratory RRi difference was calculated after implementation of the RespHRV delay for every respiratory cycle. The non-physiological RRi differences (negative values, resulting from inspiratory RRi being longer than expiratory RRi) were converted to zeros, as described by Goedhart et al. Finally, the difference was averaged across all valid respiratory cycles and used as a measure of RespHRV amplitude (RespHRVpv, ms). The absolute change in RespHRVpv before vs. after CNA was calculated (ΔRespHRVpv, ms).

The mean RRi lengths during inspiration (insRRi, ms) and expiration (expRRi, ms) were calculated by averaging the values over all valid respiratory cycles, taking into account the RespHRV delay.

The RespHRV efficiency was expressed as the percentage of (i) all inspirations accompanied by RRi shortening (short-RRi inspirations, %); (ii) all expirations accompanied by RRi lengthening (long-RRi expirations, %). RRi shortening or lengthening was detected by subtracting the last RRi from the first RRi within a search window (as defined above, including the RespHRV delay), with positive values indicating RRi shortening and negative values indicating RRi lengthening.

### 2.4. Cardiac Baroreflex Sensitivity Assessment

The CardioSeries Software (danielpenteado.com, accessed: 1 August 2025) was used to calculate spontaneous cardiac baroreflex sensitivity (BRS) with the sequence method [17]. In brief, all sequences of three or more cardiac cycles characterized by concordant changes in RRi and SBP (either RRi prolongation with SBP rise or RRi shortening with SBP fall) were extracted, and the average slope of all the regression lines constructed for all the sequences was taken as a measure of BRS (ms/mmHg). Only the sequences with r ≥ 0.8 were included in the analysis. The thresholds for changes in RRi and SBP were set at 2.5 ms and 0.5 mmHg, respectively. The absolute change in BRS before vs. after CNA was calculated (ΔBRS, ms/mmHg).

### 2.5. Parasympathetic Cardioneuroablation

CNA involves percutaneous, transcatheter ablation of parasympathetic ganglia and fibers embedded within the pericardiac fat that supply the sinus node. This is achieved by applying radiofrequency energy from the right and left atria at the anatomical locations of those ganglia. The procedure employs a 3D electroanatomic system (CARTO, Biosense Webster, Irvine, CA, USA) to accurately map the heart chambers and identify the sinus node, which is carefully preserved during ablation. In the current study, CNA was performed based on anatomical landmarks, as previously described [2,18,19]. Due to the variable anatomy of the parasympathetic system and differences in how energy penetrates the pericardiac tissue, complete vagal denervation of the sinus node is rarely achieved [2].

## 3. Results

We studied 11 patients (5 males and 6 females) with a mean age of 36.8 ± 14.1 years and a mean body mass index of 23.6 ± 3.4 kg/m^2^. Except for one case of mild hypertension treated with an angiotensin-converting enzyme inhibitor, no other medical conditions were present, and participants were not taking any cardiovascular drugs.

### 3.1. Changes in RespHRV Following CNA

**Figure 1:**
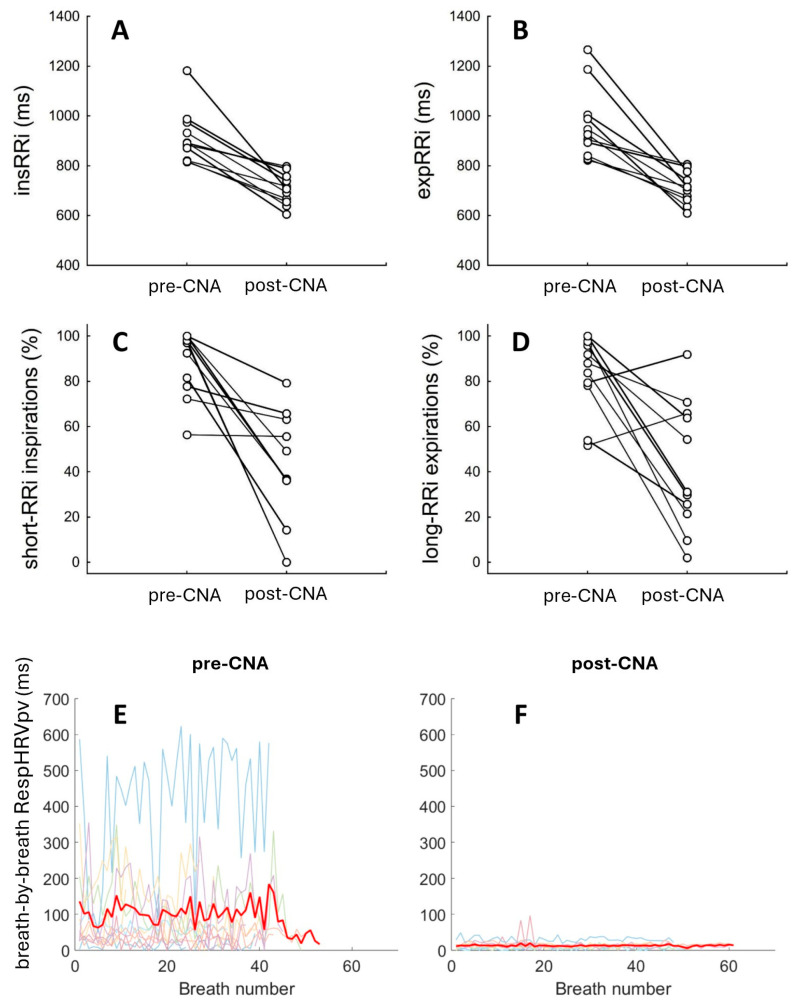
Individual data points (N = 11) on (**A**–**D**): pre- and post-CNA values of the mean RRi length during the inspiratory phase (**A**) or expiratory phase (**B**); the percentage of inspirations accompanied by RRi shortening (**C**); the percentage of expirations accompanied by RRi lengthening (**D**); (**E**,**F**) pre- and post-CNA series of the breath-by-breath RespHRVpv values. The thickened red line on panels (**E**,**F**) indicates the averaged breath-by-breath RespHRVpv.

CNA led to increased HR during both inspiratory and expiratory phases. We observed a significant decrease in all RespHRV indices (RespHRVpv, short-RRi inspirations, long-RRi expirations) after CNA. Notably, there was no change in RespHRV delay following CNA (see Table 1 for details). ΔRespHRVpv was −90.9 ± 111.7 ms. Individual data points for RespHRV efficiency and amplitude are shown in Figure 1.

### 3.2. Changes in BRS Following CNA

CNA led to a marked reduction in BRS (18.7 ± 17.4 vs. 2.9 ± 1.2 ms/mmHg, *p* = 0.010) with a ΔBRS of −16.5 ± 17.3 ms/mmHg.

### 3.3. Relationships Between Changes in RespHRV and BRS

**Figure 2:**
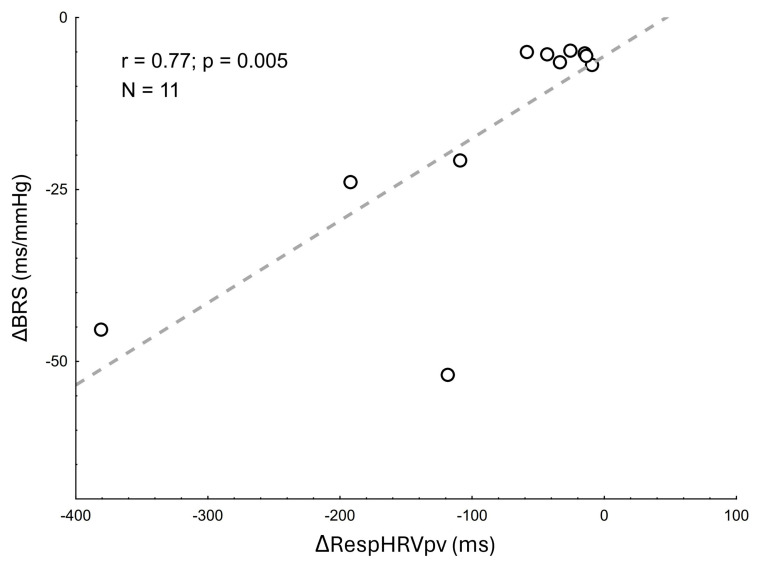
Scatterplot illustrating the correlation between pre- vs. post-CNA changes in BRS and RespHRVpv.

We observed a strong relationship between ΔBRS and ΔRespHRVpv (r = 0.77, *p* = 0.005, Figure 2). No relationships were found between the changes in BRS gain and other RespHRV indices after CNA.

## 4. Discussion

The major new findings of our study are as follows: (1) CNA reduces all measures of RespHRV, including its amplitude and efficiency; (2) the change in RespHRV amplitude after CNA is related to concurrent changes in cardiac baroreflex gain; (3) parasympathetic denervation does not influence the time delay between respiration and HR response.

Given that CNA does not interfere with the sympathetic control of the sinus node [2], the significant reduction in all measured aspects of RespHRV indicates direct involvement of parasympathetic fibers in the pathophysiology of RespHRV. Furthermore, our findings challenge the role of stretch-activation of cation nonselective ion channels within the sinus node [10] as during CNA, the sinus node region was preserved from ablation through precise electroanatomic mapping (as described in the Section 2). On the other hand, ablation of the atrial musculature and autonomic ganglia/fibers (including sympathetic ones), even at some distance from the sinus node, could potentially affect the magnitude of stretch within the sinus node. Thus, the contribution of stretch-activation cannot be completely dismissed based on our findings.

The observations regarding changes in RespHRV following CNA have not been previously reported to the best of our knowledge. However, Jungen et al. [20] described a reduction in RespHRV amplitude after catheter-based pulmonary vein isolation (PVI), which, by proximity, affects ganglionated plexi supplying parasympathetic innervation to the sinus node. We expand on those findings by providing a measure of RespHRV efficiency and describing changes in RespHRV in relation to alterations in cardiac baroreflex control. It should also be noted that the CNA used in our study specifically targeted the parasympathetic ganglia of the heart. Therefore, the effect on RespHRV dynamics might have been considerably more prominent compared to PVI, where neuromodulation could be seen as a less predictable side effect.

Do our results provide additional evidence regarding the origin of RespHRV? CNA targets the common endpoint of two mechanisms, potentially involved in maintaining RespHRV. These are (1) the central HR oscillator and (2) the peripheral baroreflex-mediated pathway. While a reduction in RespHRV measures alone does not differentiate between these possibilities, the significant relationship observed between the changes in BRS gain and RespHRVpv supports the involvement of the baroreflex in RespHRV. However, it is also possible that the central pathway was affected by CNA to the same extent as the baroreflex-mediated pathway (assuming that they share the same efferent fibers), leaving the question of the direct role of the brainstem HR oscillator unanswered. Thus, based on the data from our study involving ablation of the pericardiac tissue only, it is impossible to directly establish the role of the central vs. baroreflex-mediated pathway in maintaining RespHRV. That would be possible only if the separate parts of the autonomic system were targeted in isolation. Perhaps employing baroreflex activation therapy (BAT) [21] in future research could help resolve this issue.

The most important aspect of our study is its clinical implications. As reported by Kulakowski et al. [11], more than one in ten individuals after CNA experience impaired exercise tolerance. While the overall acceptance rate of CNA remains very high due to the elimination of syncopal episodes [22], the subjective perception of worsened exercise capacity is clinically concerning and has not yet been explained. Considering the documented relationship between higher RespHRV and better exercise capacity—both in health and disease [12,13]—the opposite scenario is a plausible and concerning possibility, potentially related to impaired ventilation–perfusion matching [5]. As CNA becomes increasingly popular as a treatment option, we believe further studies using both subjective and objective measures of exercise tolerance should be conducted to explore the potential link between reported exercise impairment after CNA and changes in RespHRV indices. Those measures should include key spiroergometry parameters such as peakVO_2_ and VE/VCO_2_ slope. Based on an important report by Ferreira et al. [23] (the AFTER-CA study), the degree of disruption within the parasympathetic innervation of the heart is expected to lessen with time. Nonetheless, the AFTER-CA study assessed the effect of PVI, where durable vagal denervation was not the major target of the procedure, as it is with CNA. In a recent report focused solely on CNA, the degree of parasympathetic denervation (assessed with atropine) decreased significantly over long-term follow-up (from 67.1% just after CNA to 28.4% at 12 months). This was mirrored by similar changes in the HRV and BRS [24]. It is very plausible that RespHRV indices would follow the same pattern over time.

The delay between respiratory events and the related changes in HR remained unchanged after CNA. This supports the idea that the speed of signal transmission through the pathophysiological chain behind RespHRV is unaffected, as ablation mostly impacts the number of viable parasympathetic fibers rather than their conductive properties.

One of the novel findings of our study is reduced RespHRV efficiency after CNA. This may partly be due to the elimination of RespHRVpv in some subjects, which makes analyzing RespHRV efficiency less reliable. However, such cases were rare in our sample, where the median RespHRVpv remained at about 20% of the pre-CNA values. Importantly, RSA efficiency was quantified using a direction-based, threshold-free approach, in which RRi shortening or lengthening was defined solely by the sign of the RRi difference within a physiologically defined respiratory window, thereby avoiding arbitrary amplitude cut-offs that could bias efficiency estimates under conditions of reduced RSA amplitude. As illustrated in Supplementary Figure S1, although CNA leads to a marked narrowing of RRi-difference distributions toward zero, their preserved non-random structure supports the robustness of RSA efficiency as a measure of disrupted cardiorespiratory coupling. Again, based on our findings, we cannot make a definitive statement about the pathophysiology of RespHRV efficiency beyond the involvement of the vagal cardiac system. Keeping in mind the previously postulated central origin of cardiorespiratory coupling [9], one could expect that CNA might also interfere with brainstem regulatory pathways. As CNA directly affects not only the efferent but also the afferent parasympathetic fibers [25], central modulation remains a possibility.

Our study is not without limitations. Firstly, long-term measurements of exercise capacity (e.g., from spiroergometry) could have shed additional light on the clinical role of reduced RespHRV after CNA; however, given the small sample size of our cohort and the low reported incidence of worsened exercise tolerance post-CNA [11], we doubt that it would have been robust enough to draw firm clinical conclusions. Second, the small sample size could have affected the negative results reported in our study. Nonetheless, the positive ones (such as reductions in most of the RespHRV indices) hold a very low probability of type I error, as indicated by *p*-values < 0.01.

## 5. Conclusions

In this study, for the first time in humans, we found direct evidence from an invasive experiment that the parasympathetic system plays a major role in the origin of RespHRV. The significant decrease in both the amplitude and efficiency of RespHRV after CNA raises concerns about the physiological effects of depriving or significantly reducing this important, evolutionarily ingrained mechanism. Further studies are needed to explore the long-term impact of CNA on cardiorespiratory control.

## Figures and Tables

**Table 1:** Respiratory heart rate variability before vs. after CNA (N = 11).

	Before CNA	After CNA	*p*-Value
insRRi, ms	891 [820–975]	708 [655–757]	0.003
expRRi, ms	927 [840–1005]	715 [664–777]	0.003
RespHRVpv, ms	57 [30–131]	13 [7–16]	0.003
short-RRi inspirations, %	97.0 [77.8–100.0]	36.0 [14.3–63.2]	0.003
long-RRi expirations, %	88.0 [78.1–97.6]	31.1 [21.4–65.8]	0.008
RespHRV delay, ms	550 [350–620]	410 [360–900]	0.721

Data are presented as median with lower and upper quartiles; *p*-value for before vs. after CNA comparison with Wilcoxon’s test is shown. Abbreviations: insRRi, mean RR interval during inspiration; expRRi, mean RR interval during expiration; RespHRVpv, respiratory heart rate variability amplitude as assessed with the peak–valley method; short-RRi inspirations, percentage of all inspirations accompanied by RR interval shortening; long-RRi expirations, percentage of all expirations accompanied by RR interval lengthening.

## Data Availability

Data will be made available upon reasonable request. For that purpose, please contact the corresponding author, Piotr Niewinski, at piotr.niewinski@umw.edu.pl.

## Supplementary Materials

The following supporting information can be downloaded at: https://www.mdpi.com/article/10.3390/jcm15010382/s1, Figure S1. Three-dimensional histograms illustrating the distribution of RR interval differences (ΔRRI) for each individual before (panel A) and after cardioneuroablation (CNA, panel B).

## Abbreviations

The following abbreviations are used in this manuscript:

BAT	Baroreflex activation therapy
BRS	Baroreflex sensitivity
CNA	Cardioneuroablation
ECG	Electrocardiogram
expRRi	Mean RR during expiration
HR	Heart rate
insRRi	Mean RR during inspiration
long-RRi expirations	Percentage of all expirations accompanied by RR interval lengthening
peak VO_2_	Peak oxygen consumption
PVI	Pulmonary vein isolation
RespHRV	Respiratory heart rate variability
RespHRVpv	Respiratory heart rate variability amplitude as assessed with the peak–valley method
SBP	Systolic blood pressure
short-RRi inspirations	Percentage of all inspirations accompanied by RR interval shortening
VE/VCO_2_ slope	Minute ventilation/carbon dioxide production slope
