# Supplementary material for: Cardioneuroablation Acutely Affects the Amplitude and Efficiency of Respiratory Heart Rate Variability
Source: J Clin Med. 2026 Jan 5;15(1):382. doi: 10.3390/jcm15010382 (PMC12786548; doi:10.3390/jcm15010382)
Supplement: Supplementary file 1 [file jcm-15-00382-s001.zip › jcm-4032341-supplementary.pdf]

Cardioneuroablation Acutely Affects the Amplitude and Efficiency of Respiratory Heart Rate Variability

Piotr Niewinski, Stanislaw Tubek, Krzysztof Nowak, Krystian Josiak and Bartłomiej Paleczny

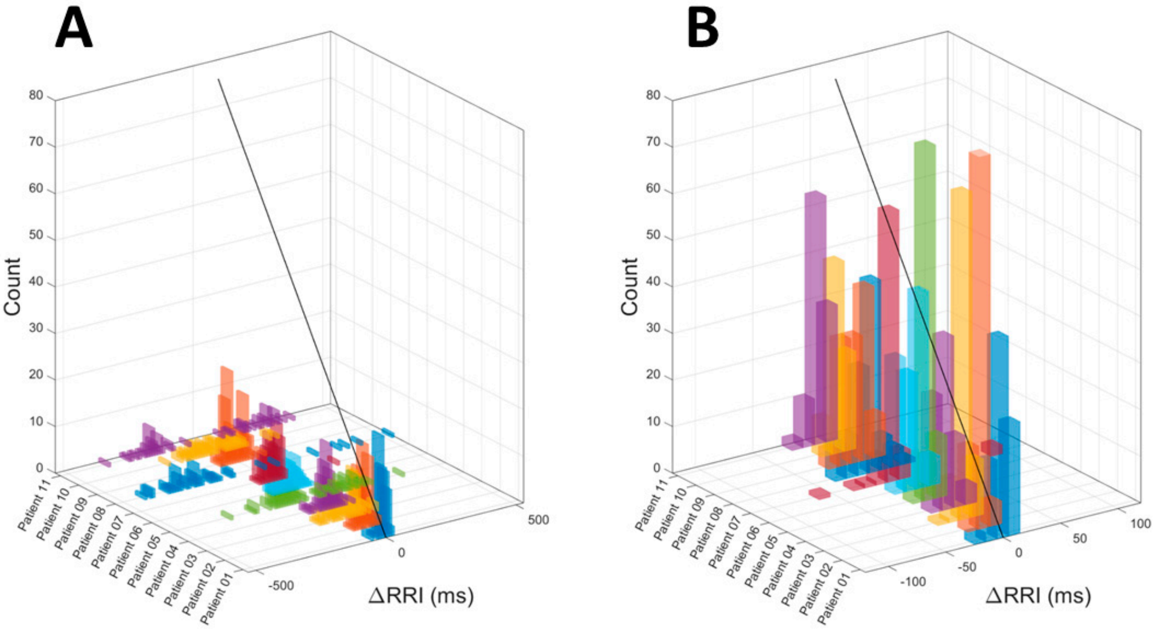

Supplementary Figure S1. Three-dimensional histograms illustrating the distribution of RR interval differences (ΔRRI) for each individual before (panel A) and after cardioneuroablation (CNA, panel B). ΔRRI was calculated as RRI(first) minus RRI(last) within the respiratory phase (combined data from inspirations and expirations). Positive ΔRRI values indicate RR-interval shortening, whereas negative values indicate RR-interval lengthening. Each subject is displayed as an individual, semi-transparent histogram (Patient 01 – Patient 11). Note that the X-axis scale differs between the pre- and post-CNA panels. ΔRRI values were binned using a bin width of 10 ms. The figure demonstrates a marked post-CNA reduction of ΔRRI distributions toward zero, while preserving non-random structure, supporting the robustness of the direction-based RSA efficiency metric even in conditions of reduced RSA amplitude.
